# *Rosa26* Locus Supports Tissue-Specific Promoter Driving Transgene Expression Specifically in Pig

**DOI:** 10.1371/journal.pone.0107945

**Published:** 2014-09-18

**Authors:** Qingran Kong, Tang Hai, Jing Ma, Tianqing Huang, Dandan Jiang, Bingteng Xie, Meiling Wu, Jiaqiang Wang, Yuran Song, Ying Wang, Yilong He, Jialu Sun, Kui Hu, Runfa Guo, Liu Wang, Qi Zhou, Yanshuang Mu, Zhonghua Liu

**Affiliations:** 1 Laboratory of Embryo Biotechnology, College of life science, Northeast Agricultural University, Harbin, China; 2 State Key Laboratory of Reproductive Biology, Institute of Zoology, Chinese Academy of Sciences, Beijing, China; 3 Graduate University of the Chinese Academy of Sciences, Beijing, China; University of Florida, United States of America

## Abstract

Genetically modified pigs have become a popular model system in fundamental research, agricultural and biomedical applications. However, random integration often result in unstable expression of transgene and unpredictable phenotypes. The *Rosa26* locus has been widely used to produce genetic modified animals with high and consistent expressing of transgene in mouse, human and rat, as it can be targeted efficiently and is not subject to gene-silencing effects. Recently, the first case of reporter gene targeting pigs in porcine *Rosa26* (*pRosa26*) locus was reported. In the study, full sequence of *pRosa26* locus was further characterized, and the *pRosa26* promoter (pR26) was cloned and we evidenced that the new porcine endogenous promoter is suitable for driving transgene expression in a high and stable manner by avoiding DNA methylation. Furthermore, elongation factor 1a promoter (EF1a) -driven GFP reporter and Myostatin promoter (MyoP)-driven Follistatin (Fst) were successfully targeted into the *pRosa26* locusby traditional homologous recombination (HR) strategy. EF1a showed high activity and hypomethylation at the locus. And, muscle-specific promoter MyoP was activated strictly in muscle of the *pRosa26* targeted pigs, indicating *Rosa26* locus supports tissue-specific promoter driving transgene expression in its own manner. The study provided further demonstration on biomedical and agricultural applications of porcine *Rosa26* promoter and locus.

## Introduction

Genetically modified pigs hold great promise in the fields of fundamental research, agriculture and biomedicine [1]. Overexpression of transgeneis an important option to produce transgenic pigs with favorable phenotypes [2]. However, insertional mutagenesis, repeat-induced gene silencing and unknown position effect that usually happen in random integration may inhibit transgene expression [3], [4]. To fully exploit transgenic pigs, great emphasis has been placed on integration of transgene into a specific genomic locus by gene targeting.

Gene targeting technologies permit the insertion of exogenous constructs into defined genomic sites. Currently available approaches, such as zinc finger nucleases (ZFNs), transcription activator-like effector nucleases (TALENs) and clustered regularly interspaced short palindromic repeats (CRISPR)/CRISPR associated (Cas) system, are capable of inducing double-stranded breaks (DSBs) to enhance homologous recombination for producing gene-knockin pigs [5]–[8]. But, off-target DNA cleavages at unknown sites can lead to mutations that are difficult to detect and cytotoxicity happens due to the cell’s emergency response to DSBs [9]–[15]. These remain concerns for the clinical use of nuclease-based approaches. Alternatively, traditional HR strategy is likely to be devoid of aberrant genomic mutations. Traditional HR has been used for targeted introduction of transgenes and loss-of-function mutations in mouse for decades [16], [17] and gene-knockout pigs has been produced by traditional HR [1], [2]. Gene knockin by traditional HR can be achieved in embryonic stem cells (ESCs) and induced pluripotent stem cells (iPSCs), but has not succeeded in somatic cells [17]. Due to unavailability of the germline-competent stem cells, gene-knockin pig by traditional HR is not obtained yet.

Specific genomic site with high homologous combination frequency and ubiquitous transcriptional activity is also critical for success in gene targeting. The most preferred integration site used for gene targeting is the *Rosa26* locus in mouse [18]–[20]. The *Rosa26* (*Gt(ROSA)26Sor*) gene was identified originally as a ubiquitous marker in a retroviral gene-trapping screen in mouse ESCs. It expresses a non-coding RNA (ncRNA) ubiquitously in embryo and adult tissue [18]. As in mouse, the human and rat *Rosa26* loci were identified and successfully targeted by traditional HR [21], [22], suggesting gene targeting at this locus is efficient. Recently, the *pRosa26* locus has been characterized by homology search with human *Rosa26* sequences and targeted by a Cre-dependent reporter gene [23]. Now, hundreds of transgenic animals and cell lines expressing a variety of transgenes have been successfully created using the *Rosa26* locus.

Tissue-specific promoters are valuable for elucidating specific gene functions and for use in gene therapy [24], [25]. However, even in mouse, activity of tissue-specific promoters driving transgene expression at the locus has not been addressed. Manipulating gene expression in muscle is of interest for a wide array offundamental and applied research [26]. In the study, the sequence of *pRosa26* locus was further characterized, and Myostatin promoter (MyoP), a muscle-specific promoter, was targeted into the locus. We demonstrate that *Rosa26* locus supports tissue-specific promoter driving transgene expression in its own manner.

## Materials and Methods

### Animals

All the treatments of pigs in this research were approved by the Northeast Agriculture University Institutional Animal Care and Use Committee. All pigs involved in this research were raised and breed followed the guideline of Animal Husbandry Department of Heilongjiang, P.R.China.

### Identification of *pRosa26*

The mouse *Rosa26* promoter and exon 1 sequences were used as a template to search the NCBI Sscrofa 10.2 database. A highly conserved sequence (sequence similarity >88%) on porcine chromosome 13 was found as the putative *pRosa26* locus. *pRosa26* exon 1 was predicted according to the alignment of mouse *Rosa26* exon 1. Porcine ESTs mapping on the region were obtained by BlastN (NCBI). RT-PCR were performed using primers aligned within the predicted exon 1 (forward: 5′CCGCCTAGAGAAGAGGCTGTGC3′) and the ESTs to clone *pRosa26* ncRNA. 2 exons flanking 1 intron were amplified with the primer based on the EST sequence of EW546160 (reverse: 5′CCAGCTGCCTCCTGTGATTACC3′). To reveal the full-length of *pRosa26*, we performed 5′ and 3′ RACE using 5′-full RACE Kit and 3′-full RACE Core Set Ver. 2.0 (Takara). 5′ RACE primers (5′CTAGCCGAGGCTCTCTGAGGAGCC3′ and 5′GCACAGCCTCTTCTCTAGGCGG3′) were designed according to exon 1 and 3′ RACE primers (5′GGAGAAAGATAGGTTCAAGTTGAG3′ and 5′GGTAATCACAGGAGGCAGCTGG3′) were designed according to EW546160. Gap sequence was amplified by nested PCR using outer primer set (forward: 5′GGATCTAATTGGAGCTATAACTGCCAGC3′ and reverse: 5′GCTGAGGGTCCCAAATGCTTTG3′) and inner primer set (forward: 5′CAGATCCGAGCCACGGCTGTGAC3′ and reverse: 5′GCTGAGGGTCCCAAATGCTTTGTTTAC3′). PCR products were cloned into pMD18T (Takara) for sequencing (Invitrogen).

### Vector construction

EF1a and pR26 (with exon 1) from Yorkshire pig genome were subcloned into EGFP-C1 (Clontech) instead of CMV at 5′ AseI and 3′ Eco47III restriction sites to construct plasmids EGFP-C1/EF1a and EGFP-C1/pR26. To construct targeting vectors, 1.5 kb 5′ short arm and 3.6 kb 3′ long arm were amplified, sequenced and subcloned into PPNT6 plasmid at 5′ NotI/3′ KpnI and 5′ XhoI/3′ NheI restriction sites, respectively. The primer sequences for 5′ arm were 5′GGAGAGAGCTGCACAAGAGGGC3′ (forward) and 5′GCTATTAGCATTACGGCAACTGAGC3′ (reverse), and 5′CCAAACTAGTGTCTCTGTCTCCAGTATCTG3′ (forward) and 5′CCACTGTCCCTACACTAGAGGGTGG3′ (reverse) for 3′ arm. The sequences of IRES-GFP from TRE plasmid (addgene) and EF1a were subcloned into PPNT6 plasmid at 5′HpaI/3′ XhoI and 5′ ClaI/3′ XbaI restriction sites to construct targeting vector PPNT6/EF1a-GFP. Sequence of MyoP-Fst replacing EF1a-IRES-GFP was subcloned into PPNT6/EF1a-GFP to construct PPNT6/MyoP-Fst targeting vector.

### Cell culture and transfection

PFFs derived from E32 Congjiang minipig fetuses were transfected by liposome-mediated plasmid pEGFP-C1, EGFP-C1/EF1a and EGFP-C1/pR26, and selected by 800 ug/ml G418 for 15 days. The surviving cells were considered as positive cells and screen by Southern blot. The positive cells were cultured in DMEM+20% FBS (Gibco) and collected per 10 days from 15D to 55D for further analysis. In targeting experiment, 1×10^7^ founder PFFs from E32 Yorkshire pig fetuses were trypsinized into single and resuspended by 400 ul BTXpress High Performance Electroporation Solution (BTX), and 20 ul 1 ug/ul of the targeting vectors linearized using NotI restriction enzyme were added. Cell electroporation was induced with 1DC pulse of 240 v for 30 msec on a BTX Elector-Cell Manipulator 2001 (BTX, San Diego, CA). After electroporaton, the cells were plated on 20 10 cm dishes. 24 h later, G418 (800 ug/ml) and GANC (2 umol/L) were added into the cultures. After selection for 10 to 15 days, colonies were picked and cultured in 48-well plates using cloning cylinders, and screen by 5′ arm PCR. The forward primer for PCR screening (F: 5′CATATCGTTTGTTACGCTGGAAGG3′) is located 300 bp upstream of 5′ arm and the reverse primer (R: 5′CGTATAATGTATGCTATACGAAGTTATGC3′) is downstream of SA sequence. The expected PCR product was 1.8 kb in the correctly targeted clones. Fluorescence was detected and imaged using Nikon 70i fluorescence microscope.

### Southern blots

At least 10 ug DNA of each sample was used in Southern blot. DNA from EGFP-C1, EGFP-C1/EF1a and EGFP-C1/pR26 transfected cells were digested with Eco47III/HindIII. The hybridization probe used to detect the GFP transcription unit DNA (GFP probe) was synthesized by PCR, and the sequences of the primers were 5′GAGCAAGGGCGAGGAGCTGTTCA3′ (forward) and 5′TGCAGAATTCGAAGCTTGAGC3′ (reverse). For genotyping the cloned pigs, DNA were digested with XhoI/XbaI or HindIII/BamHI, and hybridized with KI probe or Fst probe. KI probe identifying the 3′ arm was synthesized by PCR, and the sequences of the primers were 5′GTTAGTAACTGAGCTCAGTTGCCG3′ (forward) and 5′GGGAACCACCCTACAGAGATCTG3′ (reverse). The probe used to detect the MyoP-Fst transcription unit DNA (Fst probe) was synthesized by PCR, and the sequences of the primers were 5′TTTGGTGACTTGTGACAGACAGGGTT3′ (forward) and 5′CGTTTACGTCGCCGTCCAGC3′ (reverse). The PCR products were labeled by DIG Oligonucleotide 3′-End Labeling Kit (Roche).

### Production cloned embryos and pigs

*In vitro* matured porcine oocytes were used as recipient oocytes for nuclear transfer. After 42–44 hours of maturation culture, the oocytes were treated with 1 mg/ml hyaluronidase (Sigma) to remove the surrounding granulosa and cumulus cells. Oocytes that clearly extruded a first polar body were selected as recipient cytoplasts. Cumulus-free (denuded) oocytes were enucleated by aspirating the first polar body and adjacent cytoplasm in enucleation medium with a glass pipette 25 micro-meter in diameter in TCM-199-Hepes plus 0.3% BSA and 7.5 micro-gram/ml Cytochalasin B. Donor cells were injected into the perivitelline space of enucleated oocytes. Injected oocytes were placed in fusion/activation medium (0.3 M mannitol, 1.0 mM CaCl_2_, 0.1 mM MgCl_2_, and 0.5 mM HEPES). Fusion/activation was induced with 2DC pulses of 1.2 kv/cm for 30 msec on a BTX Elector-Cell Manipulator 2001 (BTX, San Diego, CA). The embryos were cultured in porcine zygote medium-3 (PZM-3) at 38.5°C in 5% CO_2_ in air. The cleavage rate and the blastocyst rate were assessed at 48 h and 144 h after activation, and cell number was examined by staining the nucleus of cloned embryos at blastocyst stage with 5 µg/ml Hoechst 33342. For producing cloned pigs, cloned embryos after 1 day culutred were surgically transferred into the oviduct of a surrogate the day after observed estrus. An ultrasound scanner has been used to monitor the pregnancy status of the surrogates weekly after a month of implantation and the cloned piglets were delivered by natural birth.

### Q-PCR analysis

Total RNAs were extracted from each sample using the PureLink Micro-to-Midi system (Invitrogen) according to the manufacturer’s instructions, and reverse transcription was used to generate cDNAs using PrimeScript RT Reagent Kit (TaKaRa). Real-time PCR was performed using SYBR Premix Ex Taq (TaKaRa) and the 7500 Real-Time PCR System (Applied Biosystems), with the following parameters: 95°C for 30 sec, followed by 40 two steps cycles at 95°C for 5 sec and at 60°C for 34 sec. Primers for GFP were 5′TGAACCGCATCGAGCTGAAGGG3′ (forward) and 5′ACCTTGATGCCGTTCTTCTGCTTG3′ (reverse). The primers for *pRosa26* were 5′GGCTCCTCAGAGAGCCTCGGCT3′ (forward) and 5′AGCAGCTTCCTCCAACTTCTTGGTC3′ (reverse). Fst primers were 5′CCGAATGAACAAGAAGAACAAACC3′ (forward) and 5′GTCCACCACACATGTGGAGCTG3′ (reverse). b-actin was used as a reference gene and the primer sequences were 5′AGATCGTGCGGGACATCAAG3′ (forward) and 5′GCGGCAGTGGCCATCTC3′ (reverse). The sizes of the amplification products were 110 bp for GFP, 129 bp for *pRosa26*, 229 bp for Fst and 88 bp for b-actin. For each cDNA sample, both target and reference genes were always amplified independently in triplicate on the same plate and in the same experimental run. A melting curve analysis showed that all reactions were free of primer–dimers or other non-specific products (data not shown). C_t_ values were calculated by the Sequence Detection System software (Applied Biosystems), and the amount of target sequence normalized to reference sequence was calculated as: 2^–△△Ct^.

### Western blots

Western blot analysis was performed by Bio-X Vision Biological Technology Co., Ltd. For Western blot analysis, total proteins were isolated from the samples by homogenization in lysis buffer (50 mM Tris-HCl, pH 7.5, 150 mM NaCl, 1% Triton X-100, 0.25% sodium deoxycholate, and complete protease inhibitor cocktail (Roche). The concentration of proteins was measured by Bradford reagent (Sigma), separated on 10% SDS-PAGE gels and transferred to Immobilon-P membranes (Millipore). After blocking in 5% low-fat milk in PBST (0.1% Tween 20 in PBS) for 1 h, the membranes were incubated with GFP antibody (1∶500, Santa Cruz Biotechnology), Fst antibody (1∶500, Santa Cruz Biotechnology) or mouse b-actin antibody (1∶2000, Santa Cruz Biotechnology) overnight at 4°C. After washing in PBST, the membranes were incubated in goat anti-rabbit antibody conjugated with horsera dish peroxidase (1∶5000) for 1 h, followed by three washes in PBST. The signals were detected by ECL Chemiluminescent kit (Amersham Pharmacia Biotech, Arlington Heights).

### Flow cytometry analysis

The transgenic positive cells expressing GFP driven by EF1a, CMV and pR26 were cultured and proliferated. The fluorescence intensities of the cells were analyzed in a FACS Calibur (Becton-Dickinson). The argon laser was tuned at 488 nm, and fluorescent cells were evaluated with a 525 nm band-pass filter. To set the parameters for flow cytometry analysis, non-transfected fibroblast cells were used as a negative control.

### Bisulfite sequencing

Bisulfite modification was performed on 0.3 ug of DNA from each sample using the EZ DNA Methylation-Gold Kit (Zymo research), according to the instruction manual. PCR primers to amplify the promoters were designed by Methyl Primer Express Software v1.0, which was also used to predict CpG islands and CpG sites in the sequences. A 273 bp sequence in pR26 containing one CpG island with 38 CpG sites was amplified using the primer pair: 5′GTGAGTTTTTGAGTGTAGG3′ (forward) and 5′CCAAAACACAACCTCTTCTCTA3′ (reverse). A 307 bp sequence in EF1a containing two CpG islands with 44 CpG sites was amplified using the primer pair: 5′GTTTGAGTTGAGGTTTGGTT3′ (forward) and 5′CCATTTTAAACTCCCTACAACA3′ (reverse). A 297 bp sequence in MyoP containing one CpG island with 11 CpG sites was amplified using the primer pair: 5′TTATTGGTGTGGTAAGTTGTTTT3′ (forward) and 5′CAACACTCCTCCTTACTCAATT3′ (reverse). The amplification of bisulfited-modified DNA was performed using Hot start Taq™ polymerase (TaKaRa), with the following conditions: 94°C for 5 min, followed by 40 three steps cycles at 94°C for 30 sec, 52°C for 30 sec and at 72°C for 1 min. The PCR products were separated on 1% agarose gels and purified, followed by sequencing (Invitrogen). The presence of a cytosine residue after bisulfite treatment shows that the cytosine residue was protected from bisulfite modification by methylation. Methylated and non-methylated CpG dinucleotides of each clone are illustrated with closed and open circles, respectively. At least ten clones were sequenced and analyzed for each sample.

### Statistical analysis

Statistical analysis was performed using SPSS 13.0 for MicroSoft Windows. Data are shown as mean ± SD. One-way ANOVA was used to assess differences between groups. The Duncan method was employed for pairwise comparison and followed by Bonferroni correction. P<0.05 (two-tailed) was considered as statistically significance.

## Results

### Identification of porcine *Rosa26*

**Figure 1:**
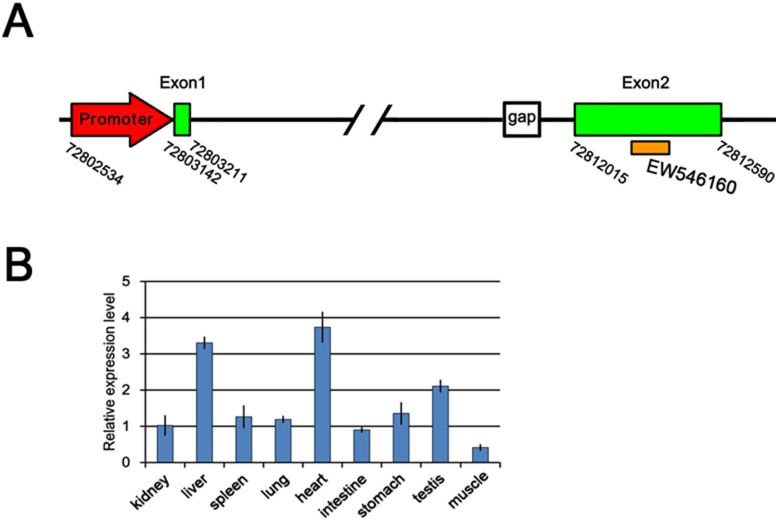
Identification of the *pRosa26* locus. (A) A diagram of the *pRosa26* locus on chromosome 13. (B) Expression of *pRosa26* ncRNA in various tissues relative to b-actin by Q-PCR. Error bars are mean±SD.

Using the mouse *Rosa26* promoter and exon 1 sequences (761 bp) as a template to search the NCBI Sscrofa 10.2 database, we located a highly conserved region (sequence similarity >88%) on porcine chromosome 13 (Figure S1). A large number of ESTs and transcripts map to the genomic region by screen of the Ensemble database. To get the ncRNA of *pRosa26*, we designed primers aligned within the predicted exon 1 and ESTs locating to the region to perform RT-PCR. 2 exons flanking 1 intron were amplified with the primers based on the EST sequence of EW546160, and the *pRosa26* ncRNA was transcribed in the opposite strand of one *ThumpD3* gene (Figure S2). 3′ and 5′ RACE were performed and a 646 bp transcript with termination signal was identified, suggesting at least we got the full-length of one transcript variant of *pRosa26* (FigureS3 and Figure 1A). Q-PCR analysis showed that the ncRNA was expressed in a wide variety of adult tissues (Figure 1B). Part of the sequence of intron 1 is not available in the NCBI Sscrofa 10.2 database. To facilitate gene targeting, we supplied 422 bp sequence for the gap by nest PCR using flanking primer sets (Figure S3).

### Activity of *pRosa26* promoter driving transgene expression

**Figure 2:**
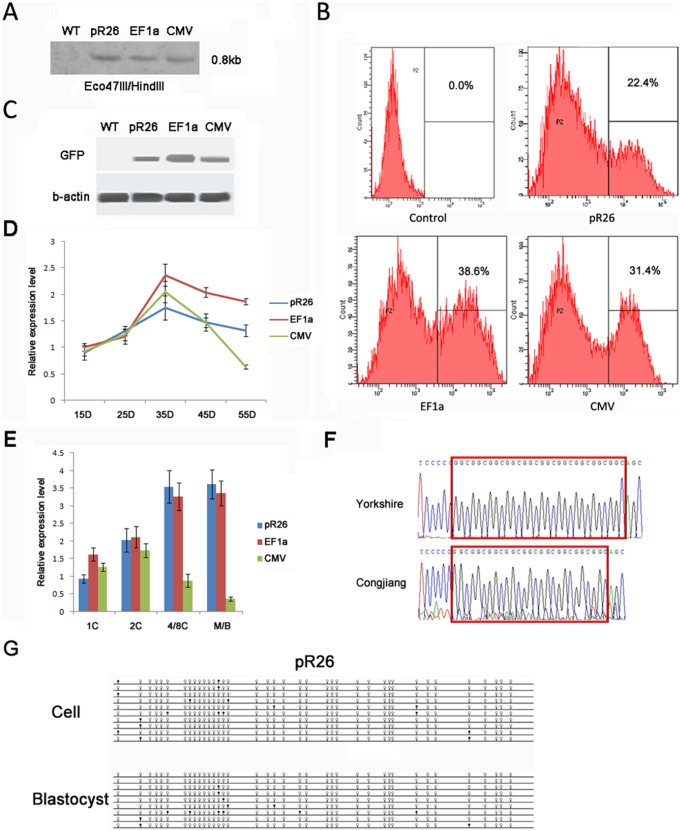
GFP expression driven by the *pRosa26* promoter. (A) Southern blot of the transgenic cell lines. Expected bands of 0.8 kb were detected after Eco47III/HindIII digestion. (B) Flow cytometry analysis of the transgenic cell lines. (C) GFP expression in the transgenic PFFs detected by Western blot. (D) GFP expression in transgenic PFFs over a long term culture up to 55D detected by Q-PCR. (E) GFP expression in transgenic cloned embryos detected by Q-PCR. (F) pR26 sequence of Congjiang minpig and Yorkshire pig. There is a lack of GGC in pR26 sequence of Congjiang minpig compared to Yorkshire pig. (G) DNA methylation status of pR26 in transgenic PFFs and cloned blastocysts. The methylation status was detected by the bisulfite sequencing. Methylated and non-methylated CpG dinucleotides of each clone are illustrated with closed and open circles, respectively.

To determine whether pR26 can highly and consistently dirve gene expression just like the mouse promoter, we compared expressions of GFP reporters driven by pR26, CMV and EF1a in porcine fetal fibroblasts (PFFs) and embryos. pR26, CMV and EF1a-driven GFP were transfected into PFFs and that was comfirmed by Southern blot (Figure 2A). Flow cytometry and Western blot analysis showed GFP expressions were incomparable levels among the three cell lines (Figure 2B and C). Stable expressions were observed in pR26-driven GFP transgenic PFFs up to 55D and cloned embryos using the PFFs as donor cells, and the pattern was highly similar to EF1a-driven GFP (Figure 2D and E). Promoter activity is negatively regulated by DNA methylation [27]. For bisulfite sequencing analysis of pR26 that drives GFP reporter, pR26 of Congjiang minipig with a lack of GGC was used to transfect PFFs of Yorkshire pig (Figure 2F). We found that pR26 could remain hypomethylated inPFFs and cloned embryos (Figure 2G). These data suggest that pR26 is suitable for driving transgene expression in a high and stable manner.

### Activity of ubiquitous promoter in the *pRosa26* locus

**Figure 3:**
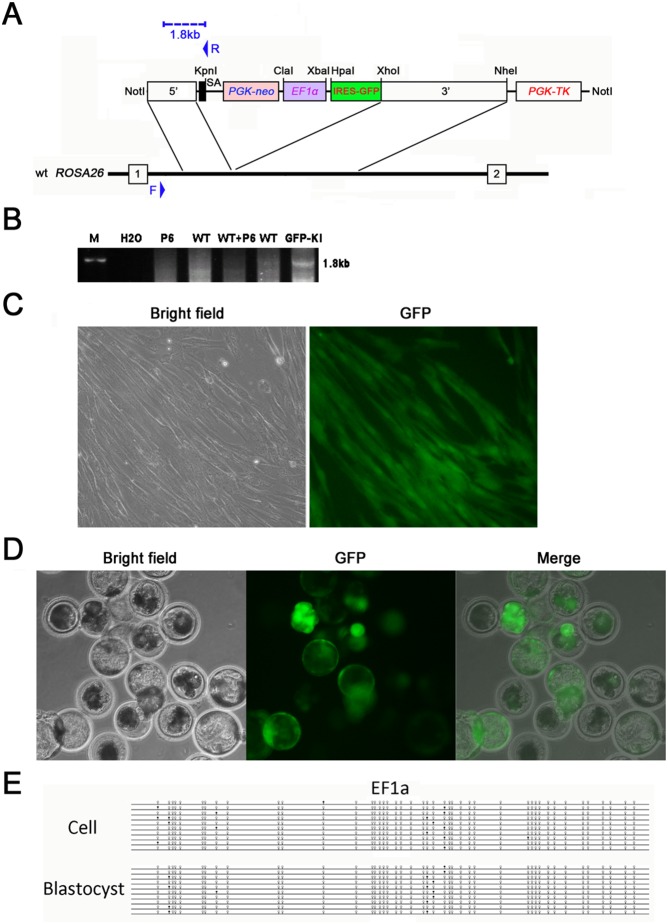
EF1a-GFP targeting in the *pRosa26* locus. (A) Schematic representive of the EF1a-GFP targeting vector and a segment of the *pRosa26* locus. SA, splice acceptor. The blue dashed line indicates the band size (1.8 kb) in the 5′ arm PCR analysis using F and R primer set. (B) 5′ arm PCR analysis of targeted cell clone. M, DNA marker. P6, PPNT6 plasmid. WT, wild type DNA. (C) GFP expression in correctly targeted cells (200×). (D) GFP expression in cloned blastocysts from targeted cells (100×). (E) DNA methylation status of EF1a in *pRosa26* targeted cells and cloned blastocysts. The methylation status was detected by the bisulfite sequencing. Methylated and non-methylated CpG dinucleotides of each clone are illustrated with closed and open circles, respectively.

To check whether the *pRosa26* locus allowed widely and high activity of ubiquitous promoter, we targeted EF1a-driven GFP into the locus by traditional HR strategy. The targeting vector contains a 1.5 kb 5′ short arm and a 3.6 kb 3′ long arm, together spanning 5.1 kb of intron 1 of the *pRosa26* locus (Figure 3A). PFFs were electroporated with the linearized targeting vector and selected by G418 (800 ug/ml) and GANC (2 umol/L). After 10 to 15 days selection, 404 clones were expanded and screened by genotyping PCR. One correctly targeted clone (GFP-KI) was identified by 5′ arm PCR analysis (Figure 3B) and GFP expression (Figure 3C), and used as donor cells to construct cloned embryos. The targeting efficiency was 0.25% (Table 1). GFP-KI cloned embryos developed to blastocyst in a rate with no significant difference compared with control group and expressed high level of GFP (Table 2 and Figure 3D). Bisulfite sequencing analysis demonstrated hypomethylated status of EF1a in GFP-KI PFFs and blastocysts (Figure 3E). These data indicate that the *pRosa26* locus has an advantage in maintenance of promoter activity by avoiding epigenetic silencing.

**Table 1:** The targeting efficiency of GFP and Fst.

Groups	Expanded cell clones	Positive targeted clones screened by 5′ arm PCR	Targeting efficiency (%)
GFP	404	1	0.25
Fst	516	3	0.58

**Table 2:** The development of GFP-KI cloned embryos *in* *vitro.*

Groups	Embryos	Fusion (%)	Cleavage (%)	Blastocyst (%)	Cell No. ofblastocyst	GFP positiveblastocyst (%)
Control	220	70.0±2.6 (n = 154)	85.7±5.0 (n = 132)	22.1±4.5 (n = 34)	31.4±3.4	–
GFP-KI	244	65.2±3.0 (n = 159)	83.3±3.0 (n = 132)	25.7±7.2 (n = 34)	35.5±4.9	83.4±3.0 (n = 28)

Note: Different superscripts mean significant difference (p<0.05).

### Activity of tissue-specific promoter in the *pRosa26* locus

**Figure 4:**
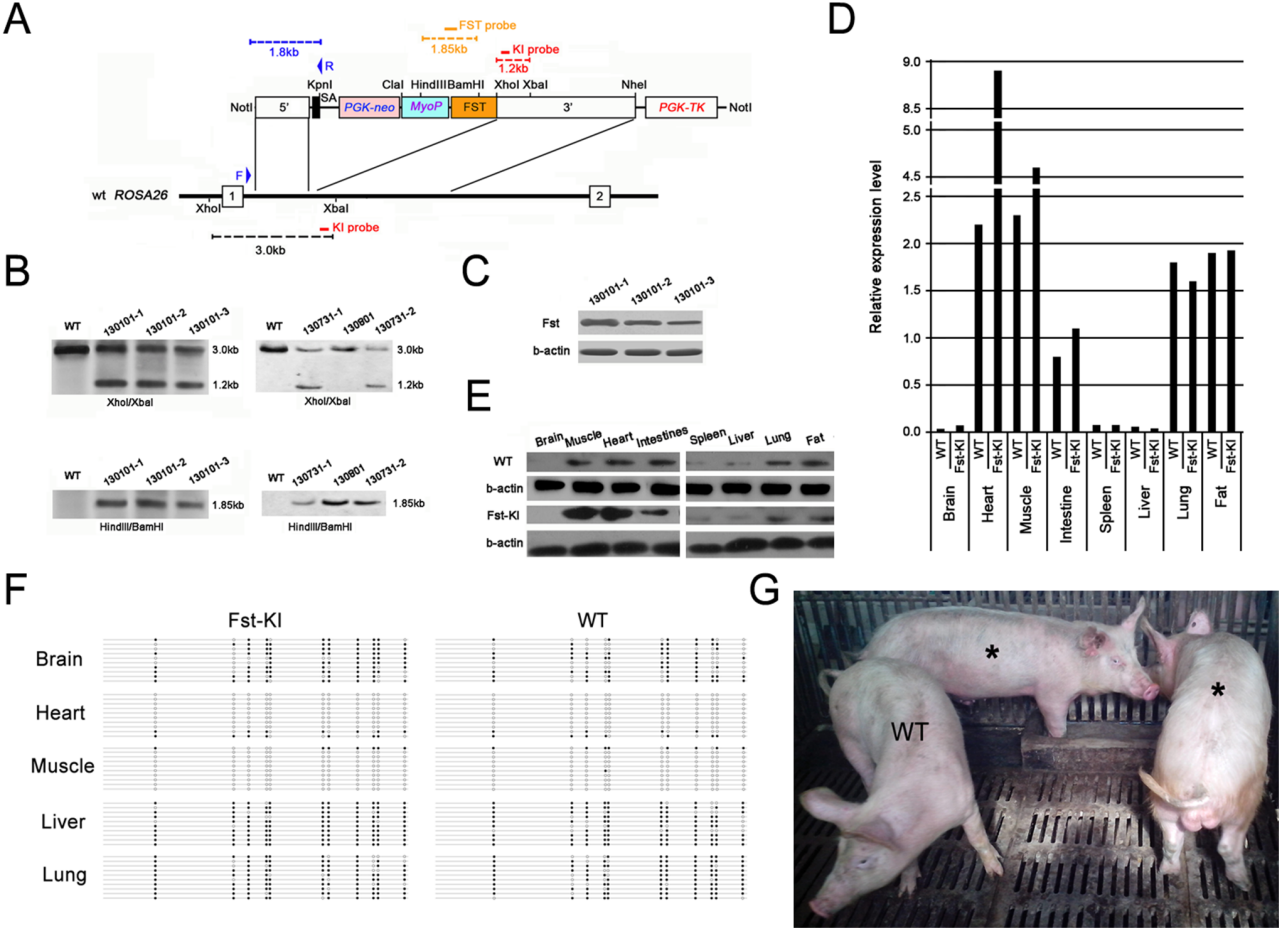
MyoP-Fst targeting in the *pRosa26* locus. (A) Schematic representive of the MyoP-Fst targeting vector and a segment of the *pRosa26* locus. SA, splice acceptor. The red and black dashed lines indicate the knockin (1.2 kb) and WT (3.0 kb) band sizes expected after XhoI/XbaI digestion in the Southern blot. The yellow dashed line indicates the Fst band size (1.85 kb) expected after HindIII/BamHI digestion in the Southern blot. The blue dashed line indicates the band size (1.8 kb) in the 5′ arm PCR analysis using F and R primer set. (B) Southern blot of cloned pigs. 5 correctly targeted pigs were confirmed. (C) Fst expression in ear of 3 Fst-KI pigs by Western blot. (D) Fst expression relative to b-actin in various tissues of Fst-KI pig by Q-PCR. (E) Fst expression in various tissues of Fst-KI pig by Western blot. (F) DNA methylation status of MyoP in various tissues of Fst-KI pig. (G) Fst-KI pigs. 130731-1 and -2 are marked with *. WT, wild type.

*Rosa26* locus enables high activity of ubiquitous promoter driving transgene expression of transgene [28], [29], however, the activity of tissue-specific promoter targeted into the locus has not been addressed. In the study, we targeted MyoP-driven Fst (MyoP-Fst) into the *pRosa26* locus. MyoP-Fst expression cassette was subcloned into the targeting vector, replacing EF1a-IRES-GFP (Figure 4A). After electroporation and selection, 516 clones of PFFs were expanded and screened by 5′ arm PCR. 3 out of 516 clones were identified as correctly targeting clones and used as donor cells to construct cloned embryos. The targeting efficiency was 0.58% (Table 1). A total of 3206 cloned embryos were transferred into 16 recipient mothers. 3 recipients were pregnant and 6 cloned piglets were obtained (Table 3). By Southern blot analysis, 5 correctly targeted pigs (Fst-KI) with the integration of a single copy of the targeting vector were confirmed, except one cloned pig (130801) from cell line Fst-KI130103 proved to be untargeted (Figure 4B). Western blot demonstrated Fst expression in ear of 3 Fst-KI stillbirths (Figure 4C). Q-PCR (Figure 4D) and Western blot (Figure 4E) analysis showed remarkedly higher expression of Fst in MyoP-specifically active tissues of heart and muscle in Fst-KI pig (130101-1) comparing with WT pig. Corresponding with that, hypomethylated status of MyoP was observed in these tissues (Figure 4F). And in other tissues such as brain, intestine, spleen, liver, lung and fat, MyoP was low active and hypermethylated. These results indicate that *pRosa26* locus supports MyoP driving transgene expression in a muscle-specific manner. Until now, 2 (130731-1 and 130731-2) of 5 correctly targeted pigs are still alive (Figure 4G).

**Table 3:** The development of Fst-KI cloned embryos *in* *vivo.*

Cell lines	No. of recipients	No. of transferredembryos	No. of pregnantrecipients	No. of born	No. of targetingpositive
Fst-KI120528	6	1238	1	3	3 (130101-1 to -3)
Fst-KI130112	5	1008	1	2	2 (130731-1 and -2)
Fst-KI130103	5	960	1	1 (130801)	0

Note: 130731-1 and 130731-2 pigs are still alive.

## Discussion

The number of genetically modified pigs has dramatically increased in recent years. However, common obstacles have been lack of desired phenotypes [1]–[4]. This study describes the identification and characterization of the porcine *Rosa26* promoter and locus, and demonstrates the activities of ubiquitous and tissue-specific promoters at the locus, providing an important advancement for genetical modification inpig.

Consistent with previous report [23], we confirmed that porcine *Rosa26* gene located on porcine chromosome 13 and obtained a 646 bp-long transcript from the *pRosa26* locus, which is longer than the one Li et al. descrided [23]. The termination signal was found in the 3′ end of our obtained transcript, suggesting that at least we get the full-length of one transcript variant of *pRosa26*. Unlike the highly conserved promoter region of *Rosa26* gene between mouse and pig, the porcine transcript exhibited low homology with that of mouse and rat, indicating the function of *Rosa26* gene may be non-conservative. Actually, little is know of the function of *Rosa26*. It possibly works to regulate the expression of *ThumpD3* [30]. In mouse, human and rat, *Rosa26* overlaps with the *ThumpD3* gene, which is positioned in the reverse orientation downstream of *Rosa26* [18],[21],[22], however, there are two *ThumpD3* genes in pig, and *pRosa26* locates in the reverse orientation upstream of one *ThumpD3* gene and in the same orientation upstream of another *ThumpD3* gene (Figure S2).

*pRosa26* promoter, conserving functions of its homologues in mouse, human and rat, exhibited a wide activity in variety of adult tissues [31]. Furthermore, pR26 could drive transgene expression in a high and stable manner, unlike CMV, a ubiquitous promoter, which was hypermethylated resulting in a low activity, indicating the porcine endogenous promoter is not rejected in the porcine cellular contexts by epigenetic silencing. We believe that genetically modified pigs will profit from the *pRosa26* promoter.

*Rosa26* locus is an ideal site for ubiquitous expression of transgene [18], [21]–[23]. In the study, we targeted EF1a-GFP and MyoP-Fst expression cassettes into the *pRosa26* locus. Though the targeting efficiency was low (0.25% for GFP targeting and 0.58% for Fst), the gene knockin pigs were successfully obtained by traditional HR strategy. To our knowledge, this is the first study to achieve gene knockin in large animals by traditional HR, suggesting gene targeting in *Rosa26* locus is efficient. Moreover, we demonstrated that EF1a and MyoP were activated in the *pRosa26* locus. It has been evidenced that the locus appears to be an appropriate docking site for the avtivity of exogenous ubiquitous promoter [28], [29]. Consistent with that, EF1a showed high activity and hypomethylation status in the *pRosa26* locus, indicating *Rosa26* locus is as a protector against epigenetic silencing of exogenous constructs. However, MyoP was active and showed hypomethylation status in a muscle-specific manner in the *pRosa26* targeted pigs. MyoP is the promoter of Myostatin, which is restricted to muscle-specific expression and as a negative regulator of myogenesis [32], [33]. Considering that, we contribute the muscle-specific manner of MyoP at the *pRosa26* locus to specific cellular contexts and DNA methylation. It has been demonstrated that in myoblast, transcription factor MyoD upregulates myostatin promoter activity and other somatic cells, which are lack of MyoD expression, show a significant reduction of MyoP activity [32]. So, the activity of tissue-specific promoter is determined on the specific cellular contexts. Beside that, specific DNA methylation plays a crucial role in establishing and maintaining the manner of tissue-specific promoter [34]. Taken together, we demonstrate *Rosa26* locus supports tissue-specific promoter driving transgene expression in its own manner.

In summary, full sequence of the porcine *Rosa26* locus was characterized and EF1a-driven GFP reporter and Myostatin promoter-driven Follistatin were successfully targeted into the locus. These results suggest that the activity of promoter in the *Rosa26* locus depends on its own manner instead of the locus. In addition to the previous report (23), the study provided further demonstration on biomedical and agricultural applications of the porcine *Rosa26* promoter and locus.

## Supporting Information

Figure S1 **Aligment of the mouse and porcine Rosa26 sequences.** Alignment of the mouse and porcine Rosa26 sequences with the highest degree of homology (sequence similarity >88%). The top arrow denotes the 5′ start of the mouse Rosa26 transcript, and the bottom arrow indicates the start of the 5′ porcine transcript.
(TIF)

Figure S2 **Porcine ESTs and transcripts neighboring the pRosa26 locus.** pRosa26 locus and multiple aligment plot of porcine ESTs and transcripts. This region contains both the pRosa26 locus and the neighboring genes that have also been found in mouse, human and rat. EW546160 used to design primers to clone pRosa26 is marked with yellow shadow. In mouse, human and rat, Rosa26 overlaps with the ThumpD3 gene, which is positioned in the reverse orientation downstream of Rosa26, however, in pig, there are two ThumpD3 genes, and pRosa26 locates in the reverse orientation upstream of one ThumpD3 gene and in the same orientation upstream of another ThumpD3 gene.
(TIF)

Figure S3 **Sequence of pRosa26.** (A) The sequence of the pRosa26 promoter. (B) The sequence of the pRosa26 ncRNA. Termination signal is marked with red under line. Primers using in 3′ or 5′ RACE are marked with green or blue under line. Primers using in Q-PCR are marked with red bracket. (C) The gap sequence of the pRosa26 intron 1.
(TIF)
