# Supplementary figures and images for: Rosa26 Locus Supports Tissue-Specific Promoter Driving Transgene Expression Specifically in Pig
Source: PLoS One. 2014 Sep 18;9(9):e107945. doi: 10.1371/journal.pone.0107945 (PMC4169413; doi:10.1371/journal.pone.0107945)

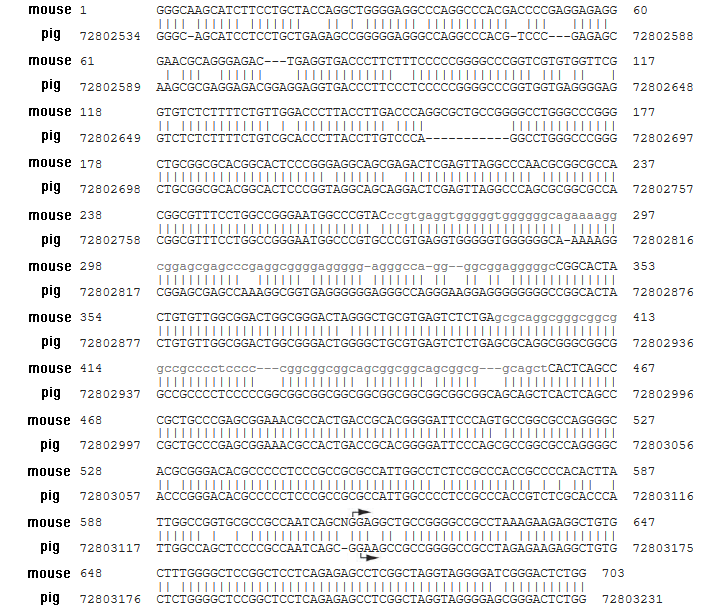

Supplement: Figure S1 — Aligment of the mouse and porcine Rosa26 sequences. Alignment of the mouse and porcine Rosa26 sequences with the highest degree of homology (sequence similarity >88%). The top arrow denotes the 5′ start of the mouse Rosa26 transcript, and the bottom arrow indicates the start of the 5′ porcine transcript. (TIF) [file pone.0107945.s001.tif]

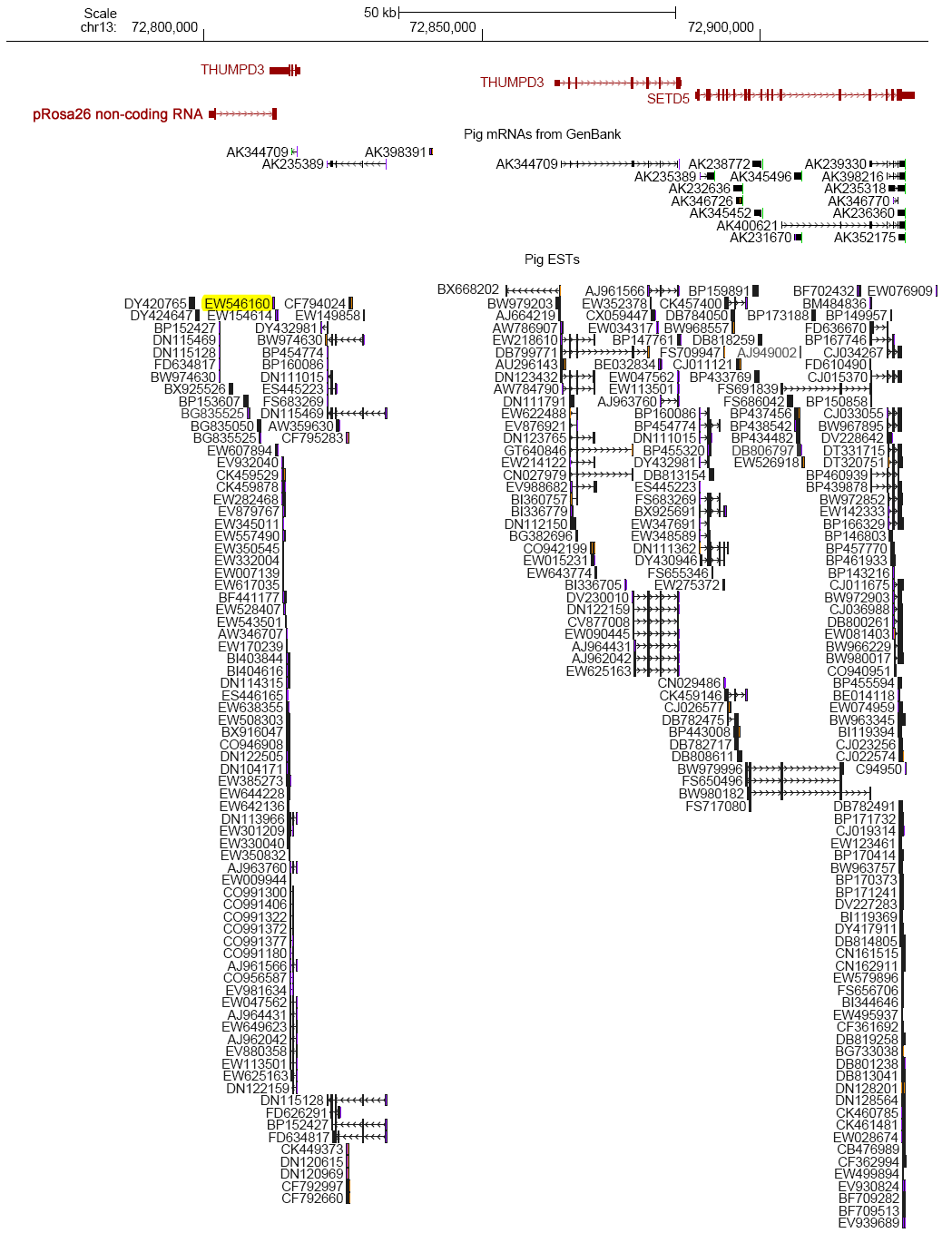

Supplement: Figure S2 — Porcine ESTs and transcripts neighboring the pRosa26 locus. pRosa26 locus and multiple aligment plot of porcine ESTs and transcripts. This region contains both the pRosa26 locus and the neighboring genes that have also been found in mouse, human and rat. EW546160 used to design primers to clone pRosa26 is marked with yellow shadow. In mouse, human and rat, Rosa26 overlaps with the ThumpD3 gene, which is positioned in the reverse orientation downstream of Rosa26, however, in pig, there are two ThumpD3 genes, and pRosa26 locates in the reverse orientation upstream of one ThumpD3 gene and in the same orientation upstream of another ThumpD3 gene. (TIF) [file pone.0107945.s002.tif]

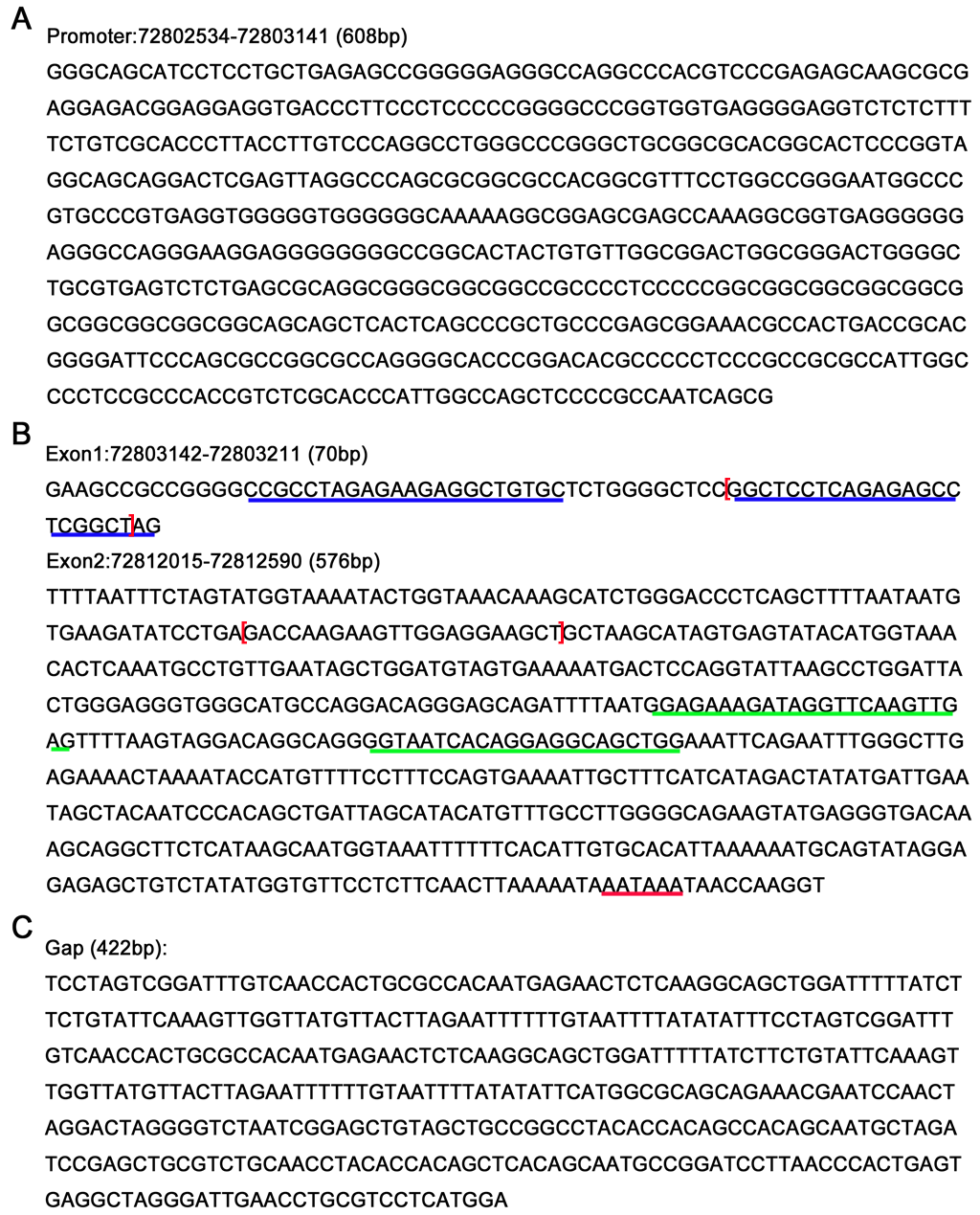

Supplement: Figure S3 — Sequence of pRosa26. (A) The sequence of the pRosa26 promoter. (B) The sequence of the pRosa26 ncRNA. Termination signal is marked with red under line. Primers using in 3′ or 5′ RACE are marked with green or blue under line. Primers using in Q-PCR are marked with red bracket. (C) The gap sequence of the pRosa26 intron 1. (TIF) [file pone.0107945.s003.tif]
